# Safety and efficacy of a lifestyle intervention for pregnant women to prevent excessive maternal weight gain: a cluster-randomized controlled trial

**DOI:** 10.1186/1471-2393-13-151

**Published:** 2013-07-16

**Authors:** Kathrin Rauh, Elisabeth Gabriel, Eva Kerschbaum, Tibor Schuster, Ruediger von Kries, Ulrike Amann-Gassner, Hans Hauner

**Affiliations:** 1 Else Kroener-Fresenius-Center for Nutritional Medicine, Chair of Nutritional Medicine, Technische Universität München, Freising-Weihenstephan, Germany; 2 ZIEL - Research Center for Nutrition and Food Sciences, Technische Universität München, Freising-Weihenstephan, Germany; 3 Competence Center for Nutrition, Freising, Germany; 4 Institute for Medical Statistics and Epidemiology, Klinikum rechts der Isar, Technische Universität München, Munich, Germany; 5 Institute of Social Pediatrics and Adolescent Medicine, Ludwig-Maximilians-Universität München, Munich, Germany; 6 Else Kroener-Fresenius-Center for Nutritional Medicine, Klinikum rechts der Isar, Uptown München Campus D, Technische Universität München, Munich, Germany

**Keywords:** Gestational weight gain (GWG), Lifestyle intervention, Pregnancy, Obesity prevention and management, Feasibility study

## Abstract

**Background:**

Excessive gestational weight gain (GWG) is associated with short- and long-term health problems among mothers and their offspring. There is a strong need for effective intervention strategies targeting excessive GWG to prevent adverse outcomes.

**Methods:**

We performed a cluster-randomized controlled intervention trial in eight gynecological practices evaluating the feasibility and effectiveness of a lifestyle intervention presented to all pregnant women; 250 healthy, pregnant women were recruited for the study. The intervention program consisted of two individually delivered counseling sessions focusing on diet, physical activity, and weight monitoring. The primary outcome was the proportion of pregnant women exceeding weight gain recommendations of the Institute of Medicine (IOM). Secondary outcome variables were maternal weight retention and short-term obstetric and neonatal outcomes.

**Results:**

The intervention resulted in a lower proportion of women exceeding IOM guidelines among women in the intervention group (38%) compared with the control group (60%) (odds ratio (OR): 0.5; 95% confidence interval (CI): 0.3 to 0.9) without prompting an increase in the proportion of pregnancies with suboptimal weight gain (19% vs. 21%). Participants in the intervention group gained significantly less weight than those in the control group. Only 17% of the women in the intervention group showed substantial weight retention of more than 5 kg compared with 31% of those in the control group at month four postpartum (pp) (OR: 0.5; 95% CI: 0.2 to 0.9). There were no significant differences in obstetric and neonatal outcomes.

**Conclusions:**

Lifestyle counseling given to pregnant women reduced the proportion of pregnancies with excessive GWG without increasing suboptimal weight gain, and may exert favorable effects on pp weight retention.

**Trial registration:**

German Clinical Trials Register DRKS00003801.

## Background

Obesity is one of the major public health concerns in the world and has reached epidemic proportions. Since 1980, the worldwide prevalence of obesity has more than doubled [1]. In Germany, the prevalence of obesity - defined as a body mass index (BMI) ≥ 30 kg/m^2^ - has increased considerably in the past three decades [2], and has reached a rate of about 23% in adults [3]. This epidemic also affects younger adults, including women of reproductive age [4]. Maternal overweight or obesity is linked to maternal, fetal, neonatal, and childhood morbidity, which has been extensively reviewed [5-9].

Not only prepregnancy obesity, but also gestational weight gain (GWG) has been found to be an independent risk factor for maternal and fetal outcomes such as: gestational diabetes [10,11], cesarean delivery [12-16], and accelerated fetal growth; indicating a strong relationship between higher GWG and increased risk of large for gestational age (LGA) births [13,14,16,17]. Maternal weight retention pp is highly influenced by GWG [18-20], which in turn contributes to the development of obesity and related metabolic disorders in the long-term [21,22].

Recent studies have shown a positive association between GWG and an increased risk for childhood obesity [23-26], adolescent obesity [27,28], and obesity persisting into adulthood [29-31] propagating a vicious cycle of obesity [9,32,33]. Breaking this cycle at any stage with adequate intervention and prevention strategies is a big challenge. Studies done in this field have yielded mixed results [34-52]. Recent systematic or meta-analytic reviews of lifestyle interventions designed to limit GWG, report inconsistent results [53-64], thus emphasizing their heterogeneity and methodological limitations.

We performed a cluster randomized trial on a lifestyle intervention for pregnant women. Our intervention started in the mid-second trimester to reduce GWG by counseling on: diet, physical activity, and self-monitoring adherence to a personalized GWG chart. We aimed at answering the following key questions. Can the number of pregnancies with excessive GWG according to the IOM criteria be reduced by a simple lifestyle counseling compared with a control group without counseling? If “yes”, does this decrease prompt an increase in the proportion of pregnancies with suboptimal weight gain? Can the number of women with weight retention above 5 kg after four months pp be reduced?

## Methods

### Study design

The FeLIPO (**Fe**asibility of a **l**ifestyle-**i**ntervention in **p**regnancy to **o**ptimize maternal weight development) study is an open-label, prospective, cluster-randomized controlled intervention trial in a two-arm parallel group design. The study protocol was approved by the ethical committee of the Technische Universität München and registered in the German Clinical Trials Register (http://www.germanctr.de, DRKS00003801). Randomization was performed at the cluster level, i.e. gynecological practices were randomized (rather than individuals). Cluster randomization avoids spillover effects, which would have occurred if individuals were randomized and treated within the same practice.

Twenty gynecological practices were initially contacted requesting their participation in the study. All practices were accessible via public transport in the Munich area and were requested to provide a room for the counseling sessions. Eleven of the twenty practices were too busy to participate in the study, and one was disinterested in the topic of GWG. Eight gynecological practices agreed to participate and were randomly assigned to either an ‘intervention’ or ‘control group’ using a computer-generated randomization allocation table. Randomization was performed by a researcher not involved in the study design thereby preventing allocation bias. The nature of the study meant that participants and study staff were not blinded to the types of intervention.

The main study hypothesis is: counseling focusing on diet, physical activity, and weight monitoring prevents weight gain in excess of IOM recommendations. The initial power calculation was based on several criteria: a 1:1 ratio between intervention and control group; 40% of women (BMI > 18.5 kg/m^2^) gain more weight than recommended by IOM criteria (Beyerlein A, November 2009, personal communication on the basis of Bavarian perinatal data from 2007), reducing to 22% in the intervention group; 80% power and an alpha of 0.05. To achieve this, 206 pregnant women are needed. During recruitment, however, it turned out that it was easier to recruit women for the intervention group than for the control group, yielding a 2:1 ratio. We, therefore, reassessed the power calculation and increased the total sample size from 206 to 225. Considering a drop-out rate of about 10%, 250 individuals were required for the trial. We did not account for clustering in the sample size calculations.

### Study population

The study population consisted of 250 healthy, pregnant women, who were recruited from eight gynecological practices by their staff between February 2010 and August 2011 in Munich, Germany. We used the following criteria in selecting pregnant women for the study population: (1) age: older than 18 years; (2) number of live fetuses: one; (3) stage of pregnancy: pre-eighteenth week; (4) BMI: ≥ 18.5 kg/m^2^, and (5) language skills: “sufficient” German. Women were excluded if they had any condition preventing physical activity, such as cervical incompetence, placenta praevia, or persistent bleeding. Additional exclusion criteria were: diagnosis of prepregnancy diabetes and uncontrolled chronic diseases that may affect weight development like thyroid dysfunction or psychiatric diseases. All participants gave their written, informed consent for participation. All eligible women had a first appointment with the study team at their next obstetric check-up.

### Intervention

The FeLIPO intervention program consisted of two individual counseling modules given by trained researchers at the 20th and 30th week of gestation, respectively. The counseling sessions were structured and comprised the three main topics: nutrition, physical activity, and GWG monitoring. The first session lasted up to 60 minutes (min) and included the main components of the intervention. The second session (about 30 min) repeated topics from the first, but was more detailed for selected aspects in a problem-oriented manner. In addition, each counseling session included an individual component where women received personalized feedback on their nutrition and physical activity habits based on 7-day-dietary records and physical activity questionnaires. In the diet component, we explained general topics like energy balance and a healthy nutrition according to the “Deutsche Gesellschaft für Ernährung” (DGE) (German Nutrition Society) [65]. We informed participants about additional energy requirements as well as macro- and micronutrient requirements during pregnancy. The dietary intervention aimed at decreasing the intake of energy-dense foods and high-fat foods (e.g. fast food, sweets, and sugar-sweetened beverages) by substituting them with low-fat alternatives, and increasing the consumption of fruit, vegetables, and whole grain products. Another goal was improving the quality of fat consumed by increasing the amount of fish in the diet and choosing the correct fat/oil for cooking and or use as spreads. As an individual component, we analyzed the dietary records checking for individual dietary problems.

The advice on physical activity was in accordance with the current guidelines for physical activity during pregnancy from the Society of Obstetricians and Gynecologists of Canada (SOGC) [66] and the American College of Obstetricians and Gynecologists (ACOG) [67]. The following recommendations were introduced for women using the FITT (frequency, intensity, time, type) criteria: thirty minutes of moderate intensity activity on most days of the week at an appropriate heart-rate zone. Non weight-bearing or low-impact endurance exercises using the large muscle groups like walking, cycling, swimming, or aquatic exercises were proposed. Furthermore, women were provided with a list of adequate local prenatal exercise programs and advised to participate in programs like these. For each prepregnancy BMI group, the IOM’s weight gain recommendations were incorporated in weight gain charts. Each woman in the intervention group received a chart personalized according to her baseline BMI group. Participants were requested to use their charts to monitor their weight development on a weekly basis.

Thus, the intervention consisted of three main parts: (1) providing general information on a healthy lifestyle during pregnancy; (2) prompting self-monitoring of behavior by recording diet and physical activity, and self-monitoring of weight gain by using weight gain charts; and (3) setting behavioral goals based on the baseline situation (BMI, diet, physical activity) and the individual preferences of the women.

### Control group

The control group received routine prenatal care including an information leaflet with ten general statements about a healthy lifestyle during pregnancy [68], but no advice on diet or gaining weight.

### Measures and data collection

Prepregnancy weight and height was self-reported by the participants at the time of recruitment. At every antenatal visit, weight and pregnancy complications were routinely documented in the “Mutterpass” (maternity card). For every measurement, the same digital scale (a Tanita HD-327 provided by the study team) was used and the women only wore light-weight clothes. Practice staff copied maternity cards and birth records at the first postnatal visit. We received these records for data retrieval on infant anthropometrics and any complications during pregnancy and delivery.

GWG was defined as self-reported prepregnancy weight and weight at the last obstetric visit prior to delivery; the latter was recorded on the maternity cards. LGA and SGA refer to infants whose birth weights were greater than and less than the 90^th^ and 10^th^ percentile adjusted for gestational age, respectively. All participants were offered a free standardized two hour oral glucose tolerance test (OGTT) between the 24^th^ and 28^th^ week of gestation, to screen for gestational diabetes mellitus (GDM). Tests were performed and interpreted according to the 2010 clinical practice guidelines of the German Society of Gynecology and Obstetrics [69]. A follow-up interview (phone call or e-mail) was arranged four months pp for both groups to record self-reported maternal weights for monitoring of weight retention. A substantial weight retention was defined when a woman’s weight was more than 5 kg greater than her prepregnancy weight four months after delivery. This cut off point was chosen as 5 kg weight retention represents a substantial shift in weight, and data analyses suggest this cut off point predicts later obesity and its consequences [70,71].

Dietary intake was assessed using 7-day dietary records, which were completed for three (16th-18th week [baseline], 26th-28th week, and 36th-38th week of gestation) and two weeks (16th-18th week [baseline], 36th-38th week of gestation) for the intervention and control group, respectively. Energy intake was calculated using the nutrition software: OptiDiet (version 5.0.0.029; Gesellschaft für optimierte Ernährung mbH – GOE). Dietary records with implausible energy intake were excluded from the statistical analysis. Underreporting of energy intake was defined using the cut off limit of Goldberg et al. (1.1 × BMR) [72]. BMR was calculated using the equation of Hronek et al. [73].

Physical activity was assessed using the IPAQ’s long version at three time intervals in both groups: 16th-18th week [baseline]; 26th-28th week; and 36th-38th week of gestation [74]. The questionnaires were analyzed according to the guidelines for data processing and analysis [75]. The volume of activity was computed by weighting each type of activity by its energy requirement defined in metabolic equivalents (METs) to yield a score in MET-minute. METs are defined as multiples of the resting metabolic rate, and a MET-minute is computed by multiplying the MET score of an activity by its duration in minutes. Data are presented as median MET-minutes per week (MET-min/wk) and truncated according to the IPAQ guidelines [75]. Extreme outliers were excluded by truncating the duration of each intensity exceeding 180 minutes per day to this value.

### Statistical analyses

We compared baseline characteristics between the intervention and control group examining the effectiveness of the randomization process, and identifying any potential confounding factors. We show categorical variables as numbers (percentages), and compared them using a chi-square test. Continuous variables were tested using the Mann–Whitney U test and are presented as means ± SD or median (interquartile range). Group differences in primary and secondary outcomes are illustrated as estimated marginal mean differences or odds ratios with 95% CIs. We accounted for cluster-specific (gynecological practice) effects by calculating linear mixed regression models for metric outcomes, and generalized linear mixed models for binary outcomes with cluster as the random intercept. Due to “a priori” considerations, we included age and prepregnancy BMI as adjustment variables. In the analysis of excessive/inadequate GWG, we adjusted for the prepregnancy BMI category as IOM recommendations are based on these categories. In all other analyses we used continuous BMI. We tested group effects on energy intake and physical activity using repeated measurements analysis of covariance (ANCOVA). We included baseline values as the covariate in the model, and analyzed differences in dietary and physical activity behavior between the baseline and follow-up intervals within each group using the Friedman or Wilcoxon test.

All analyses were performed using the R software package (version 2.15.1; R Foundation for Statistical Computing) and IBM SPSS Statistics for Windows version 19.0. A two-sided statistical evaluation of the primary study endpoint was performed at a 0.05 level of significance. We analyzed secondary study endpoints such as weight retention and obstetric outcomes in an explorative manner, and resulting p-values were not corrected for multiple testing.

## Results

### Participant flow and baseline characteristics

**Figure 1:**
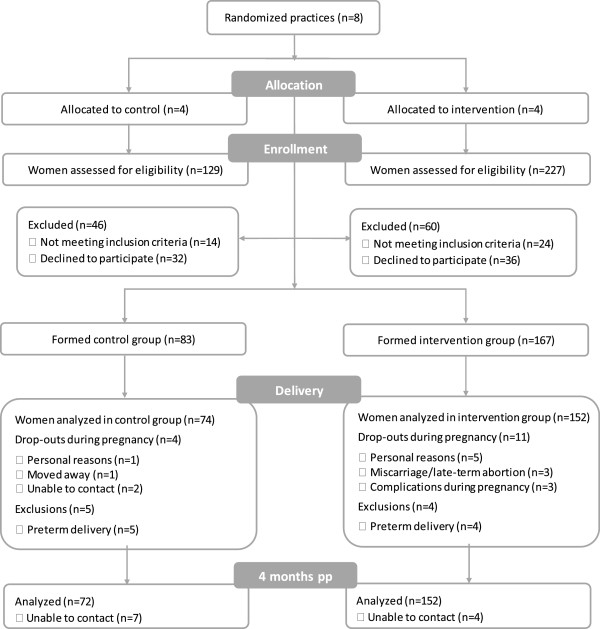
Flow diagram of the FeLIPO trial.

The participant flow in the FeLIPO study is summarized in Figure 1. Eighty three and 167 women were in the standard care and intervention group, respectively. Four (5%) and eight (5%) women in the control and intervention group, respectively, withdrew from the study due to one of the following reasons: relocation, complications during pregnancy, loss of contact, or undefined personal reasons. Two miscarriages occurred in the intervention group: one due to ruptured membranes before the first counseling session, and one due to an infection shortly after the first counseling session. One woman in the intervention group terminated her pregnancy (fetus diagnosed as a trisomy 21). One hundred and fifty six (93%) women in the intervention group attended both counseling sessions. Women who gave birth preterm (delivery before 37 weeks of gestation) were excluded from the GWG analysis. Seventy two (87% of included women) and 152 (91% of included women) of the control and intervention group, respectively, could be contacted at the fourth month follow-up consultation. Baseline characteristics and lifestyle factors for both groups are presented in Table 1. Median self-reported weight and BMI before pregnancy were, although slightly, significantly higher in the control group compared with the intervention group, which resulted from a greater proportion of overweight and obese women in the control group (31% vs. 16%). Similarly, the median measured weight at the first antenatal visit (booking) was higher among participants in the control group. When compared with self-reported prepregnancy weight, the median weight at booking was 1.9 and 1.7 kg higher in the control and intervention group, respectively. All other baseline and lifestyle-related factors were comparable between groups. We considered baseline characteristics showing significant differences between groups as adjustment variables in the analyses.

**Table 1:** Baseline characteristics of the study population

**Baseline characteristics**	**Control n = 83**	**Intervention n = 167**	**p value**^**a)**^
**Age (years)**	30.8 ± 4.9	32.2 ± 4.4	**0.010**
**Height (cm)**^**b)**^	168 ± 6	169 ± 6	0.202
**Pregravid weight (kg)**^**b)**^	63.0 (57.8 - 76.0)	62.0 (56.0 - 69.0)	**0.026**
**Pregravid BMI (kg/m**^**2**^**)**	22.8 (20.6 - 26.6)	21.7 (19.9 - 23.7)	**0.003**
**Weight at booking (kg)**^**c) d)**^	64.9 (59.4 - 78.1)	63.7 (58.0 - 70.8)	**0.042**
**BMI at booking (kg/m**^**2**^**)**^**d)**^	23.3 (21.2 - 26.8)	22.2 (20.7 - 24.3)	**0.008**
**Gestational age at booking (wk)**^**d)**^	7 (6 - 8)	9 (8 - 11)	**<0.0001**
**Pregravid BMI category**			
**Normal weight**	57 (68.7)	140 (83.8)	**0.009**
**Overweight**	15 (18.1)	20 (12.0)	
**Obese**	11 (13.3)	7 (4.2)	
**Parity**			
**0**	53 (63.9)	110 (65.9)	0.385
**1**	23 (27.7)	50 (29.9)	
**≥ 2**	7 (8.4)	7 (4.2)	
**Country of birth**			
**Germany**	68 (81.9)	140 (83.8)	0.704
**Others**	15 (18.1)	27 (16.2)	
**Marital status**			
**Married**	48 (57.8)	96 (57.5)	0.958
**Single/Divorced**	35 (42.2)	71 (42.5)	
**Graduation**			
**None/General secondary school**	7 (8.4)	8 (4.8)	0.101
**Intermediate secondary school**	24 (28.9)	30 (18.0)	
**High school/Grammar school**	15 (18.1)	32 (19.2)	
**University degree**	37 (44.6)	97 (58.1)	
**Working status**			
**Full time**	52 (62.7)	103 (61.7)	0.502
**Part time**	17 (20.5)	43 (25.7)	
**Not working**	14 (16.9)	21 (12.6)	
**Smoking**			
**No**	77 (92.8)	157 (94.0)	0.706
**Yes**	6 (7.2)	10 (6.0)	

Data are given as mean ± standard deviation, median (interquartile range) or number (%).

BMI = Body mass index.

a) Mann–Whitney U test for metrical, χ2 test for categorical variables.

b) Self-reported c) Measured weight at the first antenatal visit.

d) Control n = 82; Intervention n = 163.

### Gestational weight gain

60% and 38% of women in the control and intervention group, respectively, exceeded the IOM recommendations (Table 2). This difference remained statistically significant after adjusting for age, prepregnancy BMI category, and considering cluster as a random factor (OR: 0.5; 95% CI: 0.3 to 0.9). We identified no difference between groups in the proportion of suboptimal weight gain according to IOM guidelines (19% vs. 21%, OR: 1.0; 95% CI: 0.5 to 2.1). Participants in the intervention group gained 14.1 (± 4.1) kg, which was statistically significantly less than that gained by the control group with an average of 15.6 (± 5.8) kg (Table 2). This represented a statistically significant lower weight gain of -1.7 (95% CI: -3.0 to -0.3) kg after adjusting for confounders.

**Table 2:** Gestational weight gain and postpartum weight retention in the control versus the intervention group

**Variable**	**Control**	**Intervention**	**Absolute effect size (95% CI)**	**p value**^**a)**^	**Adjusted effect size (95% CI)**	**Adjusted p value**^**b)**^
**Gestational weight gain**	n = 74	n = 152				
**Total weight gain (kg)**	15.6 ± 5.8	14.1 ± 4.1	−1.4 (−2.7 to −0.1)	**0.035**	−1.7 (−3.0 to −0.3)	**0.049**
**Women with excessive GWG (>IOM)**	44 (59.5)	58 (38.2)	0.4 (0.2 to 0.7)	**0.003**	0.5 (0.3 to 0.9)	**0.032**
**Women with inadequate GWG (<IOM)**	14 (18.9)	32 (21.1)	1.1 (0.6 to 2.3)	0.709	1.0 (0.5 to 2.1)	0.973
**Weight retention - 4th month pp**	n = 72	n = 152				
**Time of investigation (days pp)**	123 (119–129)	123 (121–128)				
**Weight retention (kg)**	3.3 ± 5.1	2.1 ± 4.3	−1.1 (−2.4 to 0.2)	0.090	−1.4 (−2.7 to −0.2)	0.070
**Women retaining > 5 kg**	22 (30.6)	26 (17.1)	0.5 (0.2 to 0.9)	**0.024**	0.5 (0.2 to 0.9)	**0.034**

GWG = Gestational weight gain, IOM = Institute of Medicine, pp = postpartum.

Data are given as means ± SD, median (interquartile range) or number (%).

Effect sizes from regression models: Continuous variables as estimated marginal mean difference (95% CIs) and categorized variables as odds ratios (95% CIs).

a) Unadjusted analysis.

b) Linear or generalized linear mixed model adjusted for cluster (random factor), age and prepregnancy BMI (in analysis of total weight gain and weight retention) / prepregnancy BMI category (in analysis of excessive/inadequate GWG).

### Postpartum weight retention

Postpartum weight retention, defined as self-reported maternal weight four months pp minus self-reported prepregnancy weight, tended to be higher in the control group (3.3 ± 5.1 kg) compared with the intervention group (2.1 ± 4.3 kg) (Table 2); the mean difference after adjusting for confounders was -1.4 kg (95% CI: -2.7 to -0.2). Only 17% of the women in the intervention group showed substantial weight retention of more than 5 kg compared with 31% of those in the control group (OR: 0.5; 95% CI: 0.2 to 0.9).

### Pregnancy and fetal outcomes

Overall, there were no statistically significant differences in obstetric and neonatal outcomes in the intervention versus the control group (Table 3). 12% and 5% of women in the control and intervention group, respectively, developed GDM or impaired glucose tolerance (IGT). However, this difference was not statistically significant before or after adjustment for confounders. Although not reaching statistical significance in either adjusted or unadjusted analyses, the cesarean delivery rate in the routine care group (42%) was greater than that in the intensive counseling group (30%). The same was true for the rates of induced deliveries (37% vs. 26%). In terms of birth weight, results indicated no statistically significant difference between groups with an average birth weight of 3,414 (± 445) g and 3,406 (± 402) g for the control and the intervention group, respectively. No differences could be detected concerning the proportion of LGA or SGA infants. There were low rates of preterm birth in both groups that were higher in the standard care group (6% vs. 3%).

**Table 3:** Pregnancy and birth related outcomes in the control versus the intervention group

**Variable**	**Control n = 79**	**Intervention n = 156**	**Absolute effect size (95% CI)**	**p value**^**a)**^	**Adjusted effect size (95% CI)**	**Adjusted p value**^**b)**^
**Birth weight (g)**	3,414 ± 445	3,406 ± 402	−8 (−122 to 106)	0.890	29 (−85 to 143)	0.637
**Birth length (cm)**	51.7 ± 2.4	51.4 ± 2.4	−0.3 (−0.1 to 0.3)	0.351	−0.2 (−1.1 to 0.8)	0.728
**GDM or IGT**^**c)**^	9 (12.2)	8 (5.4)	0.4 (0.2 to 1.1)	0.084	0.5 (0.2 to 1.4)	0.183
**Birth mode**						
**Spontaneous birth**	35 (44.3)	91 (58.3)	ref.		ref.	
**Cesarean section**	33 (41.8)	47 (30.1)	0.6 (0.3 to 1.1)	0.076	0.6 (0.4 to 1.2)	0.145
**Vacuum extraction**	11 (13.9)	18 (11.5)	0.8 (0.4 to 1.8)	0.600	0.8 (0.3 to 2.1)	0.666
**Induction of labor**	29 (36.7)	40 (25.6)	0.6 (0.3 to 1.1)	0.080	0.6 (0.3 to 1.3)	0.191
**Preterm birth**	5 (6.3)	4 (2.6)	0.4 (0.1 to 1.5)	0.169	0.3 (0.1 to 1.2)	0.088
**Infant sex**						
**female**	37 (46.8)	72 (46.2)	ref.		ref.	
**male**	42 (53.2)	84 (53.8)	1.0 (0.6 to 1.8)	0.921	1.1 (0.6 to 1.8)	0.865
**Large for gestational age (>90th percentile)**	7 (8.9)	10 (6.4)	0.7 (0.3 to 1.9)	0.495	0.8 (0.3 to 2.3)	0.702
**Small for gestational age (<10th percentile)**	3 (3.8)	6 (3.8)	1.0 (0.2 to 4.2)	0.985	1.0 (0.2 to 4.9)	0.990

GDM = Gestational diabetes mellitus; IGT = Impaired glucose tolerance.

Data are given as means ± SD or number (%).

Effect sizes from regression models: Continuous variables as estimated marginal mean difference (95% CIs) and categorized variables as odds ratios (95% CIs).

a) Unadjusted analysis; b) Linear or generalized linear mixed model adjusted for cluster (random factor), age and prepregnancy BMI.

c) In 221 (Control n = 74, Intervention n = 147) women a standardized 2 h oral glucose tolerance test was performed; GDM or IGT was defined according to the clinical practice guidelines of the German Society of Gynecology and Obstetrics from 2010 [69].

### Behavioral change

Women in the control group increased their daily energy intake from on average 2,110 (± 409) kcal at baseline to 2,328 (± 410) kcal at the end of pregnancy (p = 0.027), while women from the intervention group maintained a stable energy intake throughout their pregnancy (Table 4). When comparing the between group differences in changes from baseline to the 36-38^th^ week interval of gestation, the intervention group had a lower energy intake than the control group (mean difference: -115 kcal, 95% CI: -212 to -8, p = 0.035).

**Table 4:** Energy intake and total physical activity in the control versus the intervention group

**Variable**	**Control**	**Intervention**	**Overall effect size (95% CI)**	**p value**^**a)**^
	n = 47	n = 121		
**Energy [kcal/day]**				
**Baseline**	2,110 ± 409	2,195 ± 387	−115 (−221 to −8)	**0.035**
**26-28**^**th **^**wk**		2,174 ± 331		
**36-38**^**th **^**wk**	2,328 ± 410	2,215 ± 347		
**p value**^**b)**^	**0.027**	0.928		
	n = 55	n = 118		
**Total activity [MET-min/wk]**				
**Baseline**	3,186 (1,711 - 4,932)	2,573 (1,605 - 4,488)	207 (−304 to 717)	0.425
**26-28**^**th **^**wk**	2,826 (1,480 - 5,455)	2,529 (1,477 - 4,282)		
**36-38**^**th **^**wk**	2,232 (1,410 - 3,685)	1,968 (1,257 - 3,336)		
**p value**^**b)**^	**0.019**	0.198		

Wk = week.

Data are given as means ± SD or median (interquartile range), effect sizes as estimated marginal mean difference and 95% CIs.

a) P values for effect of intervention between groups (repeated-measurements ANOVA adjusted by baseline values).

b) Differences within groups (Friedman or Wilcoxon test).

All women significantly decreased their total physical activity during the course of pregnancy (Table 4). In the control group, median total activity significantly decreased from 3,186 MET-min/wk at baseline to 2,826 MET-min/wk at the 26-28^th^ week interval of gestation and 2,232 MET-min/wk at the 36-38^th^ week interval of gestation, while the reduction over time in the intervention group was non-significant.

## Discussion

We investigated the potential to reduce the rate of excessive weight gain during pregnancy by lifestyle counseling given to pregnant women. The intervention resulted in a lower proportion of women exceeding IOM recommendations compared with the control group without increasing inadequate GWG. Our findings are in line with earlier reports showing reductions in GWG by lifestyle intervention in pregnant women [35,40,48,51,52]. To date, diverse intervention strategies and intensities, and differences in study populations and design complicate comparisons between trials. In a meta-analysis, Streuling et al. [56] combined data from four randomized controlled trials and five non-randomized trials with a total of 1,549 women. They found a lower GWG in the intervention groups, with a mean difference of 1.2 kg. While they only selected studies with interventions combining diet and physical activity, the recent meta-analysis of Thangaratinam et al. [63] included all randomized controlled trials that evaluated any dietary or lifestyle intervention, and found a 1.4 kg reduction in GWG with any intervention compared with controls. These observed differences in weight gain are similar to those observed in our study.

However, little is known about the risk of increasing the proportion of women with suboptimal weight gain by lifestyle intervention, especially if delivered globally to all pregnant women [76]. Our results show that by using our intervention scheme reducing excessive weight gain without increasing inadequate weight gain is possible. The use of weight gain charts that mark upper and lower GWG limits, as well as individual recommendations based on nutrition and physical activity questionnaires, may be key components for effective and safe interventions that are beneficial for all pregnant women. Moreover, we evaluated the effects of our intervention on weight retention at four months pp; the lifestyle counseling significantly decreased the proportion of women retaining a substantial amount of more than 5 kg weight. These data are consistent with results obtained in other intervention trials [48,52]. A recent meta-analysis of nine observational studies concluded that gaining weight according to IOM recommendations could avoid long-term high pp weight retention [20].

Our feasibility study failed in demonstrating any statistically significant differences between the intervention and control group regarding pregnancy complications, as well as obstetric and neonatal outcomes. Also most other randomized controlled trials in this field failed in identifying any differences in such outcomes, and most including our study were inadequately powered to address these issues [38,41-43,50,51]. However, the FeLIPO study detected some favorable trends concerning the outcomes: gestational diabetes and cesarean section. These trends seem to be in line with our expectations and fit with observational data [10,11,13,15,77,78] and recent results from randomized controlled trials [40,47,49] and meta-analyses [60,62]. Nevertheless, the FeLIPO study was not designed to assess these outcomes. Further studies, adequately powered for such outcomes, are needed for thoroughly testing the effect of lifestyle counseling on pregnancy complications, and on obstetric and neonatal outcomes.

A lower energy intake may have contributed to optimizing gestational weight gain in our intervention group. In 2011, Streuling et al. performed a systematic review of observational studies with the aim of associating weight gain with dietary intake. They suggested gestational weight gain might be reduced by lower energy intake during pregnancy as supported by our data [79].

Women in both groups decreased their physical activity as pregnancy progressed. Less physical activity is common during pregnancy [80] and is mostly caused by pregnancy-related health problems like sickness; lack of energy; feeling uncomfortable due to enlarged body size; and lack of time (due to work or childcare) [[81]]. This decrease could not be prevented by our intervention. Also, most of the lifestyle intervention trials reporting data on physical activity did not observe an effect of their intervention program [34,35,37,42]. Nevertheless, women in our intervention group showed a smaller decrease in physical activity when compared to controls, which may have contributed to the effects of the intervention. Further analyses of the dietary records and physical activity questionnaires are ongoing and might provide additional insights about causes for the observed effects.

A strength of the FeLIPO study was the use of an intervention program with practical relevance that could be implemented in the health-care system for pregnant women. As intended, the intervention could be scheduled in combination with prenatal visits resulting in both high participation and low dropout rates. The possibility for a spillover effect between groups was minimized by our cluster-randomized design. However, there were some limitations in the study, for example: gestational weight gain was analyzed based on self-reported prepregnancy weight, which may have been underestimated by (especially) overweight and obese women, leading to an overestimation of total weight gain [82]. However, comparing the first measured weights recorded in maternity cards (booking) with self-reported prepregnancy weights, the latter were about 2 kg lower in both groups and the two parameters were highly correlated. This approach is widely used in this type of study where there is a lack of data concerning measured weight [60], and furthermore has yielded valid estimates [83,84]. Women in the control group were aware of participating in a trial aiming at promoting a healthy lifestyle and optimizing gestational weight gain, which may have influenced their behavior, resulting in an underestimation of the intervention effect. Significant baseline differences in prepregnancy BMI and age were identified between study groups. Although these variables were included as adjustment variables in our analyses, baseline differences between the groups contributing to the efficacy of the intervention cannot be excluded. Although the counseling sessions followed a pre-defined curriculum differences between counselors are possible. We did not account for clustering in the sample size calculations. As a further limitation, the number of women approached in the control practices was lower than in the intervention practices, which we speculate may be related to unmotivated gynecologists and practice staff recruiting participants, or to lower numbers of pregnant patients during the recruitment phase among practices randomized as the control. As practice staff and participants were not blinded to the study purpose and group allocation, referring to the control group might have influenced recruitment and participation rates, which raises the possibility of post-randomization selection. However, both groups were comparable with regard to most sociodemographic parameters. Nevertheless, larger studies are needed to confirm these results.

## Conclusions

A lifestyle counseling delivered to all pregnant women reduced the proportion of pregnancies with excessive GWG without increasing suboptimal weight gain, and may exert favorable effects on pp weight retention. The effects on other pregnancy and birth outcomes remain unclear. These findings can be incorporated into a multicenter and multidisciplinary public health project targeting maternal and fetal health. Such programs are highly justified, as intervention during pregnancy is characterized by a unique treatment adherence and appears worthwhile in view of the worldwide obesity epidemic.

## Competing interests

The authors declare that they have no competing interests.

## Authors’ contributions

HH, KR and UAG contributed to the design of the study. RvK gave scientific advice. KR, EG, EK conducted the research (enrollment of participants, lifestyle counseling, data collection, trial management). KR analyzed the data. TS provided advice on statistical analyses. KR and HH wrote the manuscript. All authors read and approved the final manuscript.

## Pre-publication history

The pre-publication history for this paper can be accessed here:

http://www.biomedcentral.com/1471-2393/13/151/prepub
